# Opposing Roles for ATP13A2 and ATP13A3 in Breast Cancer Subtype-Specific Polyamine Homeostasis

**DOI:** 10.3390/biom16020255

**Published:** 2026-02-05

**Authors:** Emily Meeus, Jan Eggermont, Sarah van Veen, Peter Vangheluwe

**Affiliations:** Laboratory of Cellular Transport Systems, Department of Cellular and Molecular Medicine, KU Leuven, 3000 Leuven, Belgium; emily.meeus@kuleuven.be (E.M.); jan.eggermont@kuleuven.be (J.E.); sarah.vanveen@kuleuven.be (S.v.V.)

**Keywords:** polyamine homeostasis, breast cancer, P5B-type ATPases, breast cancer subtypes

## Abstract

Polyamine homeostasis is essential for normal cellular function and is maintained through coordinated regulation of polyamine biosynthesis, catabolism, and transport. This balance is frequently disrupted in breast cancer, a biologically heterogeneous disease comprising distinct molecular subtypes. However, whether polyamine metabolism and transport are differentially regulated across breast cancer subtypes remains poorly defined. Here, we systematically interrogate polyamine homeostasis across representative breast cancer subtypes by integrating cell line profiling combined with analysis of publicly available patient datasets. We found subtype-associated differences across the polyamine pathway and identify polyamine transport as a key contributor to inter- and intra-subtype heterogeneity. Notably, ATP13A3 emerges as a previously unrecognized adverse prognostic marker, particularly in basal-like breast cancer, where its expression associates with proliferative and oncogenic signaling programs. In contrast, ATP13A2 shows an opposing association with patient survival, suggesting divergent functional roles for these closely related transporters. Together, our findings demonstrate that polyamine regulation in breast cancer is highly subtype dependent and highlight the importance of molecular stratification when considering polyamine-directed therapeutic strategies in breast cancer.

## 1. Introduction

**Figure 1:**
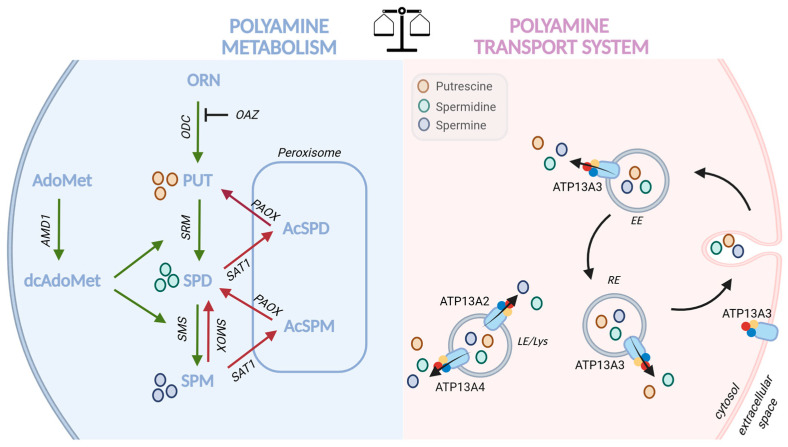
Polyamine balance is regulated through coordinated metabolic and transport pathways. AcSPD: acetyl-spermidine, AcSPM: acetyl-spermine, AdoMet: s-adenosylmethionine, DcAdoMet: decarboxylated s-adenosylmethionine, AMD1: s-adenosylmethionine decarboxylase, ORN: ornithine, OAZ: antizyme, ODC: ornithine decarboxylase, PUT: putrescine, SPD: spermidine, SPM: spermine, SRM: spermidine synthase, SMS: spermine synthase, SMOX: spermine oxidase, SAT1: spermidine/spermine N^1^-acetyltransferase 1, PAOX: polyamine oxidase, LE: late endosome, Lys: lysosome, RE: recycling endosomes, EE: early endosomes. Figure created in BioRender. van Veen, S. (2026) https://BioRender.com/45h1u63.

Polyamines, including putrescine (PUT), spermidine (SPD), and spermine (SPM), are small polycationic metabolites essential for a wide range of biological processes, including cell growth, differentiation, migration, and gene regulation. Their intracellular levels are tightly controlled through coordinated biosynthesis, catabolism, and transport [1] (Figure 1). The biosynthetic pathway relies on enzymes such as ornithine decarboxylase 1 (ODC1), S-adenosylmethionine decarboxylase (AMD1), spermidine synthase (SRM), and spermine synthase (SMS), whereas catabolic turnover is mediated by spermidine/spermine N^1^-acetyltransferase 1 (SAT1), spermine oxidase (SMOX) and polyamine oxidase (PAOX) [1]. Polyamine levels, both cytosolic and organellar, are further shaped by transport across the plasma membrane and between intracellular organelles via the polyamine transport system (PTS). Recent work has identified P5B-type ATPases as central components of the mammalian PTS [1]. Our group and others have shown that ATP13A2, ATP13A3, and ATP13A4 are expressed along the endo-/lysosomal pathway, thereby maintaining intracellular polyamine homeostasis [2,3,4]. ATP13A2 exports polyamines from the lysosomal lumen to the cytosol, thereby preventing lysosomal polyamine accumulation and maintaining cellular polyamine content. Loss-of-function variants in ATP13A2 disrupt this process and are known to cause neurodegenerative disorders [2]. ATP13A3, which localizes to the plasma membrane [4], and early/recycling endosomes, mediates cellular polyamine uptake [5], and its dysregulation has been linked to pancreatic cancer [4], head and neck cancer [6], and neuroblastoma [7]. Moreover, ATP13A3 and caveolin-1 have been shown to be regulators of polyamine uptake that control cell proliferation in pancreatic cancer [8], and vascular smooth muscle cells [9,10]. Altered ATP13A4 expression has similarly been reported in lung adenocarcinoma [11], and ovarian cancer [12], and our own work has demonstrated that ATP13A4 upregulation enhances polyamine transport activity in the MCF7 breast cancer cell line [13]. Together, these findings point to a broader role for polyamine transport dysregulation in tumorigenesis and tumor growth.

Breast cancer is among the malignancies in which polyamine dysregulation is most consistently observed. Elevated intracellular polyamine levels [14] and increased ODC1 activity [15] have been associated with enhanced tumor growth and aggressiveness. In contrast, components of the catabolic arm frequently show opposing patterns: SMOX expression is significantly reduced in breast cancer tissue compared to non-neoplastic tissue, SAT1 expression is increased, and PAOX activity is diminished and inversely correlated with tumor aggressiveness [16]. Beyond metabolic shifts, dysregulation of the PTS is increasingly recognized in breast cancer, with ATP13A4 implicated as a contributor to altered polyamine handling in breast cancer cells [13]. Such transport-related changes may further influence intracellular polyamine distribution and support tumor progression. Polyamines additionally modulate key oncogenic pathways implicated in breast cancer, including mTORC1 [17], nuclear factor kappa B [18], estrogen receptor [19], and MAPK [13,20,21] signaling, highlighting their broad functional relevance in breast cancer.

Despite these insights, most studies investigate polyamines in breast cancer as if it were a uniform disease entity. This approach overlooks the considerable biological heterogeneity among breast cancer subtypes, which differ markedly in receptor status, underlying molecular programs, clinical behavior, and therapeutic responsiveness. PAM50 is a 50-gene expression signature that classifies breast cancer into five intrinsic molecular subtypes—luminal A, luminal B, HER2-enriched, basal-like and normal-like [22,23,24]—based on tumor-intrinsic gene expression patterns, which broadly, but not exclusively, correlate with estrogen receptor (ER), progesterone receptor (PR), and human epidermal growth factor receptor 2 (HER2) status [25]. Whether these subtypes harbor unique polyamine metabolic or transport signatures remains largely unexplored. Addressing this gap is essential to understand how polyamine biology may contribute to subtype-specific tumor behavior. Here, we systematically compared polyamine homeostasis across representative breast cancer subtypes using cell line profiling combined with analysis of publicly available patient datasets. This integrated approach reveals subtype-specific differences in the polyamine pathway, identifies ATP13A3 as a previously unrecognized adverse prognostic marker, particularly in basal-like tumors, and uncovers an opposing association between ATP13A3 and ATP13A2 expression with patient survival. Together, these findings highlight the functional and clinical significance of polyamine transporters in breast cancer and establish ATP13A3 as a potential biomarker and therapeutic target in aggressive disease.

## 2. Materials and Methods

### 2.1. Cell Culture

The cell lines MCF10A (CTRL-10317), MCF7 (HTB-22), BT-474 (HTB-20), MDA-MB-453 (HTB-131), and MDA-MB-231 (HTB-26) were purchased from ATCC. The cell line SUM149PT (300609) was purchased from Cytion. Breast cancer cell lines MCF7, BT-474, MDA-MB-453, MDA-MB-231, and SUM149PT were cultured in Dulbecco’s Modified Eagle Medium (DMEM; Gibco, Waltham, MA, USA) supplemented with 10% heat-inactivated fetal bovine serum (FBS; PAN BioTech, Aidenbach, Germany), 1% penicillin/streptomycin (Merck, Rahway, NJ, USA), 1% non-essential amino acids (Merck), and GlutaMAX^TM^ (Thermo Fisher Scientific, Waltham, MA, USA). MCF10A cells were cultured in DMEM/F-12 medium (Thermo Fisher Scientific) supplemented with 5 % heat-inactivated horse serum (Merck), EGF (20 ng mL^−1^; PeproTech EC Limited, London, UK), hydrocortisone (0.5 µg mL^−1^; TCI Europe, Zwijndrecht, Belgium), cholera toxin (100 ng mL^−1^; Merck), insulin (10 μg mL^−1^; Merck), 1% penicillin/streptomycin (Merck), 1% non-essential amino acids (Merck) and GlutaMAX ^TM^ (Thermo Fisher Scientific). All cells were cultured at 37 °C with 5% CO_2_.

### 2.2. RT-qPCR

mRNA expression levels of *ATP13A2*, *ATP13A3*, and *ATP13A4* were quantified by RT-qPCR analysis. Total RNA was extracted from 1.0 × 10^6^ cells using the NucleoSpin RNA Plus Kit (740984.250, Macherey-Nagel, Düren, Germany) according to the manufacturer’s instructions. A Nanodrop spectrophotometer (Thermo Fisher) was used to measure the concentration and purity of the RNA samples. cDNA was synthesized from total RNA using the High-Capacity cDNA Reverse Transcription Kit (4368814, Thermo Fisher). Quantitative PCR was performed using SYBR Green-based detection (1725274, Bio-Rad, Hercules, CA, USA) with gene-specific primer pairs (Table 1) on a LightCycler system (Roche, Basel, Switzerland). GAPDH was used as the reference gene for normalization. Cycling conditions were as follows: 3 min at 98 °C, 50 cycles of 10 s at 98 °C, 30 s at 55 °C, 5 min at 55 °C and 5 s at 95 °C. Melt-curve analysis was performed from 55 to 95 °C to confirm amplification specificity. Mean Cq values were calculated for downstream analysis.

### 2.3. BODIPY-Polyamine Uptake

Boron dipyrromethene (BODIPY)-polyamines (BODIPY-putrescine (SCT248, Merck), BODIPY-spermidine (SCT249, Merck), and BODIPY-spermine (SCT250, Merck) were dissolved in 0.1 M MOPS-KOH (pH 7.0) to a final stock concentration of 5 mM. For the flow cytometry-based cellular uptake of BODIPY-polyamines, cells were seeded in 12-well plates at a density of 1.0 × 10^5^ cells per well. The next day, medium was pre-incubated for 15 min with 1 mM aminoguanidine to block serum amine oxidases in culture medium supplemented with FBS [26,27]. Hereafter, cells were incubated for 2 h with 1 μM BODIPY-conjugated polyamines, i.e., putrescine, spermidine and spermine. Cells were washed and resuspended in PBS supplemented with 1% BSA. Uptake was measured by recording the median fluorescence intensities of 10,000 events using the Cytek Aurora spectral cell analyzer (Cytek Biosciences, Fremont, CA, USA).

### 2.4. Western Blotting

Cells were seeded at a density of 1.0 × 10^6^ cells per 10 cm plate. The next day, cells were dissociated from the plates using TrypLE^TM^ (12604021, ThermoFisher) and lysed in RIPA buffer (89900, ThermoFisher) supplemented with EDTA-free protease inhibitor cocktail (5056489001, Merck). The protein concentration was determined using the Pierce BCA Protein Assay Kit (23225, Thermo Fisher). Proteins in cell lysates were separated on precast NuPAGE^TM^ 4–12% Bis-Tris gels (Invitrogen, Waltham, MA, USA) using MOPS running buffer (Life Sciences, Fulton, MD, USA), followed by transfer onto polyvinylidene fluoride (PVDF) membranes (1704157, Bio-Rad) according to the manufacturer’s instructions. After blocking in TBS-T (50 mM Tris-HCl, 150 mM NaCl, pH 7.5, 0.1% Tween-20 (Sigma, St. Louis, MO, USA)) supplemented with 5% non-fat dry milk, blots were incubated with primary antibodies (1/1000 dilution in TBS-T with 1% BSA) at 4 °C overnight. The following primary antibodies were used: anti-ornithine decarboxylase (ab66067, Abcam, Cambridge, UK), anti-spermine oxidase (SAB1101510, Merck), anti-spermine synthase (ab248996, Abcam), anti-spermidine synthase (ab241496, Abcam), and anti-GAPDH (G8795, Sigma Aldrich). The blots were then incubated with horseradish peroxidase (HRP)-conjugated anti-rabbit (7074, Cell Signaling, Danvers, MA, USA) and anti-mouse (7076, Cell Signaling) secondary antibodies (1/1000 dilution in TBS-T with 1% BSA) for 1–2 h at room temperature. Protein expression was detected using SuperSignal^TM^ West Pico PLUS chemiluminescent substrate (34096, Thermo Fisher Scientific) and the Bio-Rad ChemiDoc MP imaging system. Quantification was performed with the ImageJ software (version 1.54f).

### 2.5. In Silico Analysis

#### 2.5.1. The Cancer Cell Line Encyclopedia

Normalized mRNA expression data and associated metadata were obtained from the Cancer Dependency Map (DepMap) portal (https://depmap.org/portal/, accessed on 30 December 2025) using the publicly available 25Q3 release [28]. Expression of polyamine-related genes (*ODC1*, *SMS*, *SRM*, *ATP13A2*, *ATP13A3*, *ATP13A4*, *CAV1*, *SMOX*, *PAOX*, and *SAT1*) was assessed in a panel of breast cancer cell lines (MCF7, BT474, MDAMB453, MDAMB231, and SUM149PT) as well as across all breast cancer cell lines, stratified by molecular subtype. Data processing and statistical analyses were performed in R (version 4.4.3).

#### 2.5.2. TCGA-BRCA

Breast cancer gene expression and clinical data were obtained from The Cancer Genome Atlas (TCGA) Breast Invasive Carcinoma (BRCA) cohort via cBioPortal (https://www.cbioportal.org/, accessed on 30 December 2025) using the PanCancer Atlas dataset [22]. Gene expression values were log_2_-transformed (log_2_[RSEM + 1]) and molecular subtypes were assigned according to the PAM50 classification (Luminal A, Luminal B, HER2-enriched, Basal, and Normal-like). Analyses were restricted to female patients.

Expression of polyamine-related genes (*ODC1*, *SMS*, *SRM*, *ATP13A2*, *ATP13A3*, *ATP13A4*, *CAV1*, *SMOX*, *PAOX*, and *SAT1*) was evaluated across PAM50 subtypes.

Z-scores were calculated using the mean and standard deviation of Normal-like samples. Module activity scores were computed for each sample as the mean z-score of genes belonging to the same functional module [synthesis (*ODC1*, *SRM*, *SMS*), catabolism (*SMOX*, *PAOX*, SAT1), and transport (*ATP13A2*, *ATP13A3*, *ATP13A4*)].

Spearman correlation analysis was performed to assess associations between polyamine-related genes and selected proliferation-related (*MKI67* and *MCM2*) and mTOR/MAPK signaling-related genes.

Cox proportional hazards analysis was performed using overall survival as the clinical endpoint. Univariable cox models were fitted for each gene, and multivariable cox models were adjusted for patient age, tumor stage, and PAM50 molecular subtype.

For gene set enrichment analysis (GSEA), samples were stratified into high- and low-expression groups for each polyamine-related gene based on median expression levels. Differential pathway enrichment between groups was analyzed using GSEA software (version 4.4.0). All data processing and statistical analyses were performed in R (version 4.4.3).

#### 2.5.3. CPTAC-Breast Cancer

Breast cancer protein abundance and associated clinical annotations were retrieved from the Clinical Proteomic Tumor Analysis Consortium (CPTAC) cohort through cBioPortal (https://www.cbioportal.org/, accessed on 30 December 2025), using the Proteogenomic Landscape of Breast Cancer dataset [29]. Tumors were stratified in molecular subtypes based on the PAM50 classification (Luminal A, Luminal B, HER2-enriched, Basal-like). Spearman correlation analysis was used to evaluate associations between ATP13A3 and CAV1 (caveolin-1) protein expression levels.

### 2.6. Statistical Analysis

Data analysis was performed using R (version 4.4.3). Details of the statistical tests are provided in the corresponding figure legends. Bar graphs display individual points. The data are represented as mean ± SD. All experiments were repeated independently at least three times.

## 3. Results

### 3.1. Polyamine Homeostasis Differs Across Breast Cancer Subtypes

To determine whether polyamine homeostasis varies across breast cancer subtypes, we profiled a representative panel of breast cancer cell lines: MCF7 (luminal A: ER^+^/PR^+^/HER2^−^), BT474 (luminal B: ER^+^/PR^+^/HER2^+^), MDAMB453 (HER2-enriched: ER^−^/PR^−^/HER2^+^), MDAMB231 and SUM149PT (basal-like/triple-negative: ER^−^/PR^−^/HER2^−^) [30]. SUM149PT breast cancer cells harbor a deletion of a thymine residue at position 2288 of BRCA1 [31]. The non-tumorigenic epithelial cell line MCF10A served as a control. This panel enabled comparative analysis of polyamine transport and metabolic pathway components across clinically relevant breast cancer subtypes.

#### 3.1.1. Subtype-Associated Differences in Polyamine Transporters

**Figure 2:**
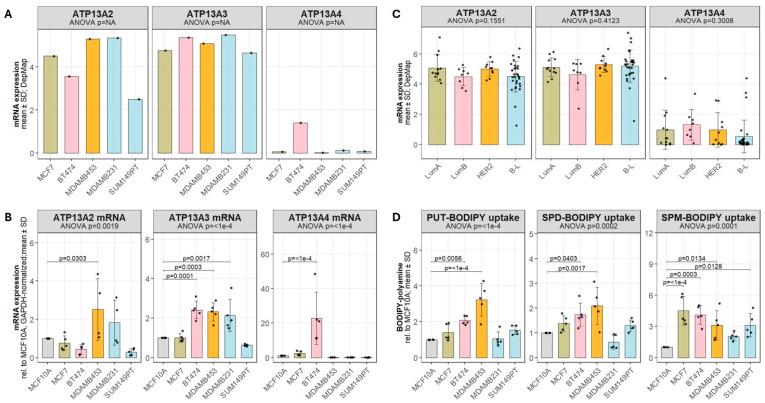
Breast cancer cell lines display different polyamine transport profiles. (**A**) mRNA levels of *ATP13A2*, *ATP13A3*, and *ATP13A4* in selected breast cancer cell lines were extracted from the Cancer Cell Line Encyclopedia (CCLE) dataset. (**B**) mRNA expression of *ATP13A2*, *ATP13A3*, and *ATP13A4* was quantified by qPCR across the same panel of cell lines. mRNA expression was normalized to *GAPDH*. The data in the bar graphs are presented as the mean ± SD of at least three independent experiments, with individual data points shown. Statistical significance was determined by one-way ANOVA followed by Dunnett’s multiple comparison test. Statistically significant *p*-values (*p* < 0.05) are depicted in the graphs. (**C**) CCLE transcriptomic data for *ATP13A2*, *ATP13A3*, and *ATP13A4* grouped by breast cancer subtype [Luminal A (LumA) n = 12, Luminal B (LumB) n = 9, HER2-enriched (HER2) n = 10, Basal-Like (B-L) n = 30 (Table 2)]. The data are presented as mean ± SD. (**D**) Cellular uptake of BODIPY-labeled putrescine (PUT-BODIPY), spermidine (SPD-BODIPY), and spermine (SPM-BODIPY) (1 µM, 2 h) was measured by flow cytometry in selected breast cancer cell lines. Statistical significance was determined by one-way ANOVA with Dunnett’s multiple comparison test. Significant *p*-values (*p* < 0.05) are indicated. The data are presented as mean ± SD.

Analysis of Cancer Cell Line Encyclopedia (CCLE) transcriptomic data revealed substantial heterogeneity in the mRNA expression of polyamine transporters across the breast cancer cell line panel. Among the P5B-ATPases, *ATP13A2* and *ATP13A3* exhibited the highest overall transcript abundance, whereas *ATP13A4* expression was generally low except for a striking elevation in BT474 cells (Figure 2A).

These expression patterns were independently validated by RT-qPCR, which confirmed significant differences in *ATP13A2* (*p* = 0.0019), *ATP13A3* (*p* ≤ 0.0001) and *ATP13A4* (*p* ≤ 0.0001) transcript levels across the cell line panel (Figure 2B). Compared to the non-tumorigenic epithelial cell line MCF10A, *ATP13A2* expression was significantly increased only in MDAMB453 cells (*p* = 0.0303), while no significant differences were observed in the other breast cancer cell lines. In contrast, *ATP13A3* expression was significantly elevated in BT474 (*p* = 0.0001), MDAMB453 (*p* = 0.0003), and MDAMB231 (*p* = 0.0017) cells, whereas *ATP13A4* expression was selectively increased in BT474 cells (*p* < 0.001) compared with MCF10A (Figure 2B). Because reliable antibodies capable of reproducibly detecting endogenous ATP13A2, ATP13A3, and ATP13A4 are currently lacking, protein-level validation in breast cancer cell lines could not be performed.

Analysis of CCLE transcriptomic data across breast cancer cell lines grouped by molecular subtype (Table 2) showed no significant subtype-associated differences in *ATP13A2*, *ATP13A3* or *ATP13A4* mRNA expression (Figure 2C). Notably, each transporter displayed substantial intra-subtype variability, underscoring that no single cell line reliably represents the full molecular diversity of its assigned subtype.

Given previous reports linking CAV1 to ATP13A3-dependent polyamine uptake [8,9,10], we next examined *CAV1* expression across the breast cancer cell line panel used in this study (Figure S1A). Analysis of CCLE/DepMap transcriptomic data revealed substantial variability in *CAV1* mRNA expression across individual cell lines. Notably, MDAMB453 cells displayed the lowest *CAV1* expression among the analyzed models while maintaining relatively high *ATP13A3* expression, consistent with a high ATP13A3/low CAV1 configuration in this specific cell line. When cell lines were grouped by molecular subtype, *CAV1* expression differed significantly across subtypes (Figure S1B), in contrast to *ATP13A2*, *ATP13A3*, and *ATP13A4*, which did not show subtype-associated differences at the transcript level (Figure 2C). Basal-like cell lines exhibited significantly higher *CAV1* expression compared with luminal A, luminal B, and HER2-enriched models, indicating that a high ATP13A3/low CAV1 transcriptional profile is not representative for all basal-like breast cancer cell lines as a group.

Because transcript levels alone do not necessarily predict transporter activity, we next assessed functional polyamine import directly using BODIPY-labeled PUT, SPD, and SPM (Figure 2D). Uptake of the three polyamines differed significantly between cell lines. MDAMB453 and BT474 displayed increased uptake of all BODIPY-polyamines compared with MCF10A, consistent with enhanced import capacity. MCF7 and SUM149PT each showed selective increases in BODIPY-SPM uptake only. In contrast, MDAMB231 did not show significant changes in uptake for any of the three polyamines. Overall, these findings indicate that polyamine import capacity is heterogeneous across breast cancer cell lines, including divergent uptake phenotypes within the basal-like/triple-negative subtype, underscoring that transporter mRNA abundance of individual isoforms alone is insufficient to infer transport activity of the combined polyamine transporter pool.

#### 3.1.2. Subtype-Associated Differences in Polyamine Metabolic Enzymes

**Figure 3:**
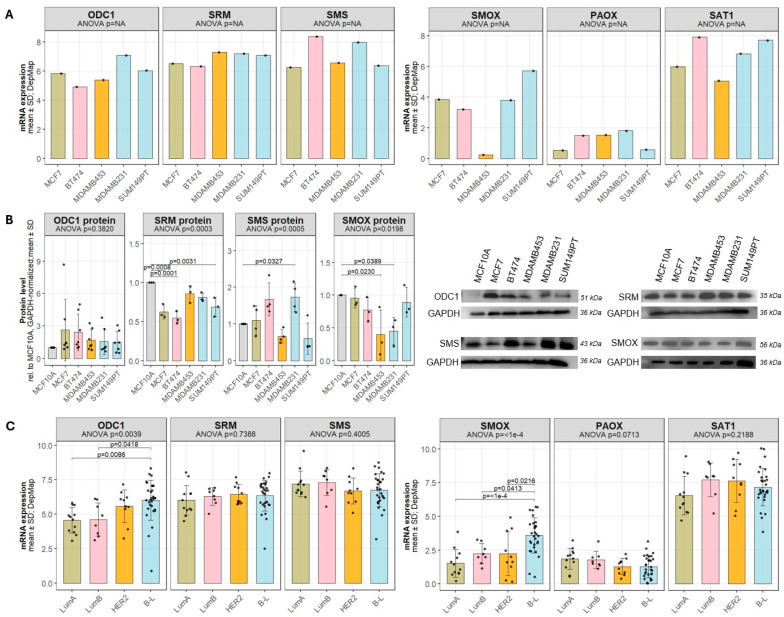
Breast cancer cell lines display different profiles of polyamine metabolism. (**A**) mRNA levels of the polyamine metabolic enzymes *ODC1*, *SRM*, *SMS*, *SMOX*, *PAOX*, and *SAT1* in selected breast cancer cell lines were extracted from the Cancer Cell Line Encyclopedia (CCLE) dataset. (**B**) Protein levels of ODC1, SRM, SMS and SMOX across the same panel of breast cancer cell lines were assessed by immunoblotting. Representative blots from at least three independent experiments are shown. Protein expression was normalized to GAPDH. Statistical significance was determined by one-way ANOVA followed by Dunnett’s multiple comparison test. Significant *p*-values (*p* < 0.05) are depicted in the graphs. (**C**) CCLE transcriptomic data for *ODC1*, *SRM*, *SMS*, *SMOX*, *PAOX*, and *SAT1* grouped by molecular subtype [Luminal A (LumA) n = 12, Luminal B (LumB) n = 9, HER2-enriched (HER2) n = 10, Basal-Like (B-L) n = 30 (Table 2)]. All data are presented as mean ± SD. Uncropped Western Blots can be found at Supplementary Figure S3.

We next examined whether polyamine metabolic enzymes show subtype-associated patterns across breast cancer models. CCLE transcriptomic data revealed that the polyamine synthesis enzymes *ODC1*, *SRM*, and *SMS* exhibited broadly similar mRNA expression levels across most cell lines. Among catabolic enzymes, *SMOX* was only reduced in the HER2-enriched line MDAMB453, *PAOX* was generally low, and *SAT1* higher across breast cancer cell lines (Figure 3A).

Immunoblotting confirmed differences at the protein level (Figure 3B). ODC1 protein levels did not differ significantly between cell lines, suggesting that the rate-limiting step of polyamine synthesis is relatively stable among the models examined. In contrast, SRM (*p* = 0.0003), SMS (*p* = 0.0005), and SMOX (*p* = 0.02) showed significant variation. Compared with MCF10A cells, SRM protein levels were significantly reduced in the luminal models (MCF7, BT474) and in the basal-like SUM149PT, whereas SMS protein levels were significantly elevated in MDAMB231. SMOX expression was significantly lower in MDAMB453 (*p* = 0.0230) consistent with transcriptomic data and indicative of a reduced catabolic capacity in this HER2-enriched cell line. Subtype-level analysis of CCLE transcriptomic data further revealed that *ODC1* and *SMOX* expression were significantly elevated in basal-like compared with luminal subtypes (Figure 3C), suggesting a more dynamic polyamine metabolic state in basal-like tumors. In contrast, expression of *SRM*, *SMS*, *PAOX*, and *SAT1* was comparable across subtypes.

Together, these analyses show that polyamine homeostasis varies across breast cancer cell lines, with some subtype-associated trends but considerable intra-subtype variability. This heterogeneity underscores the complexity of polyamine homeostasis in breast cancer and prompted us to examine patient tumors.

### 3.2. Polyamine Homeostasis Differs Across Patient-Derived Breast Cancer Subtypes

**Figure 4:**
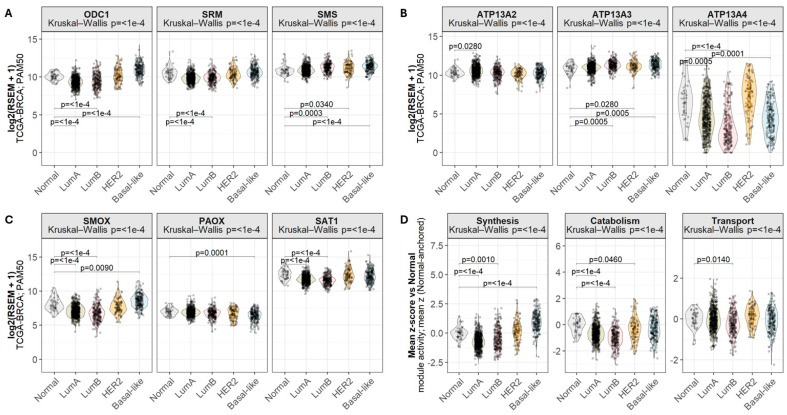
Polyamine-related gene expression in patient tumors differs across breast cancer subtypes. mRNA expression of polyamine synthesis (**A**), transport (**B**), and catabolism (**C**) genes across PAM50 breast cancer subtypes [Normal-like n = 36, Luminal A (LumA) n = 499, Luminal B (LumB) n =197, HER2-enriched (HER2) n = 78, Basal-like n = 171] was extracted from the Breast Invasive Carcinoma (TCGA, PanCancer Atlas) dataset obtained from cBioPortal. (**D**) Polyamine synthesis, catabolism, and transport across PAM breast cancer subtypes, expressed as mean gene-level z-scores normalized to PAM normal-like tissue. Statistical significance was determined by Kruskal–Wallis and Wilcoxon tests (Bonferroni post hoc-adjusted). Significant *p*-values (*p* < 0.05) are depicted in the graphs.

We next examined the mRNA expression of polyamine-related genes across PAM50 breast cancer subtypes in the TCGA-BRCA cohort to determine whether subtype-associated patterns observed in cell line analyses are recapitulated and more clearly resolved in patient tumors (Figure 4).

Among the biosynthetic enzymes, *ODC1* expression was significantly reduced in luminal A and luminal B tumors, remained largely unchanged in HER2-enriched tumors, and was significantly increased in basal-like tumors relative to normal-like breast tissue. *SMS* expression was significantly elevated in luminal B, HER2-enriched, and basal-like subtypes, whereas *SRM* expression was significantly reduced in the luminal subtypes (Figure 4A), indicating divergent regulation of individual biosynthetic steps across molecular subtypes. Subtype-dependent differences were also evident among the P5B-type ATPases. *ATP13A2* expression was significantly increased in luminal A tumors but showed no significant change in HER2-enriched and basal-like tumors compared with normal-like tissue. In contrast, *ATP13A3* expression was significantly increased in luminal B, HER2-enriched, and basal-like subtypes, whereas *ATP13A4* expression was significantly decreased in both luminal and basal-like tumors (Figure 4B), highlighting marked subtype-specific divergence among polyamine transporters. Expression of catabolic enzymes further underscored subtype-dependent differences. *SMOX* expression was significantly increased in basal-like subtypes, whereas *PAOX* expression was significantly decreased in the same subtype. In contrast, luminal subtypes showed significantly lower *SAT1* and *SMOX* expression compared with normal-like tissue (Figure 4C), consistent with reduced polyamine catabolic capacity in luminal disease.

To assess whether subtype-associated transcriptional differences in polyamine transporters are reflected at the protein level in patient tumors, we next analyzed mass spectrometry-based proteomic data from the CPTAC breast cancer cohort. In contrast to the subtype-associated transcriptomic patterns observed in TCGA, protein abundance of ATP13A2, ATP13A3, and ATP13A4 displayed substantial inter-tumor variability but did not differ significantly across PAM50 molecular subtypes (Figure S2). Notably, subtype-specific analyses in CPTAC are based on a more limited number of samples relative to TCGA transcriptomic cohorts, which reduces statistical power to detect modest subtype-associated differences in protein abundance.

We next examined CAV1 expression as a potential contextual modifier of polyamine transport. *CAV1* mRNA expression was significantly reduced across all breast cancer subtypes compared with normal-like tissue in the TCGA-BRCA cohort (Figure S1C), and this decrease was also observed at the protein level in the CPTAC cohort (Figure S1D). However, we did not detect consistent associations between CAV1 and ATP13A3 expression at either the transcript or protein level across PAM50 subtypes (Figure S1E,F), indicating that bulk tumor expression data do not support a stable coupling between CAV1 abundance and polyamine transporter expression.

Together, these data indicate that subtype-associated differences in polyamine transport inferred from transcriptomic analyses are not readily detected at the level of steady-state bulk tumor proteomics in the CPTAC cohort. Given the substantial inter-tumor variability and the more limited sample numbers available for subtype-specific proteomic analyses, the absence of strong protein-level stratification should be interpreted with caution. Nonetheless, the observed discordance between transcript and protein abundance is consistent with the possibility that regulation of polyamine uptake may occur through mechanisms beyond changes in total protein levels, including post-transcriptional regulation or alterations in membrane organization.

Finally, to determine whether these gene-level differences reflected coordinated pathway-wide transcriptional programs, we summarized polyamine synthesis, catabolism, and transport using normal-anchored module activity scores (Figure 4D). Consistent with the gene-level analyses, both synthesis and catabolism modules were significantly downregulated in luminal subtypes, whereas polyamine synthesis activity was increased in basal-like tumors. In contrast, despite overall subtype-associated variation, the polyamine transport module showed a significant decrease only in the luminal B subtype, suggesting that transcriptional regulation of polyamine transport is heterogeneous and driven by gene-specific effects rather than coordinated pathway-wide activation.

Together, these findings demonstrate that breast cancer subtypes exhibit distinct polyamine metabolic states, with basal-like tumors characterized by increased mRNA expression of biosynthetic enzymes and selective mRNA upregulation of individual polyamine transporters such as *ATP13A3*.

### 3.3. Polyamine-Related Genes Correlate Differently with Proliferation and Clinical Outcomes

**Figure 5:**
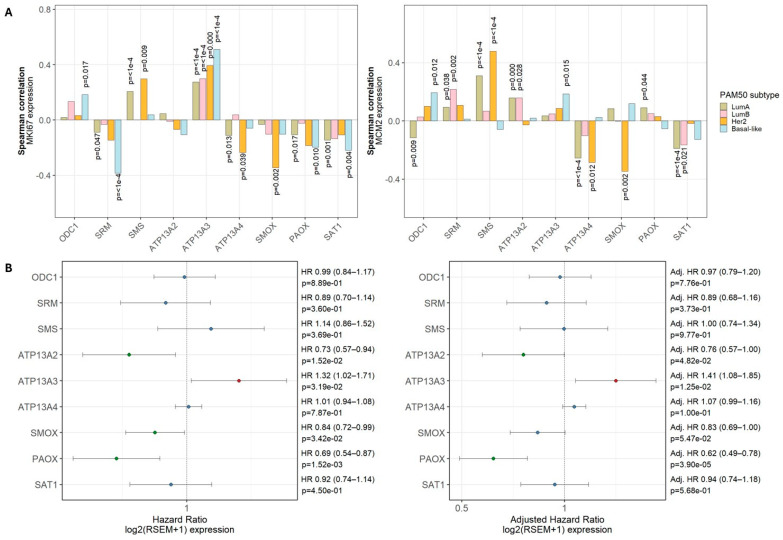
Subtype-specific association of polyamine genes with tumor proliferation and survival in breast cancer. (**A**) Spearman correlation analysis between the proliferation markers, *MKI67* (**left**) and *MCM2* (**right**), and polyamine-related genes across PAM50 breast cancer subtypes. Significant *p*-values (*p* < 0.05) are shown in the graphs. (**B**) Univariable Cox proportional hazards models assessing the association between polyamine-related genes and overall survival (**left**), and multivariable Cox proportional hazards models adjusting for stage, age, and subtype (**right**).

To assess whether polyamine-related genes are linked to tumor proliferation and clinical outcome, we quantified their associations with the proliferation markers *MKI67* and *MCM2* [32] at the transcript level across PAM50 subtypes (Figure 5A) and evaluated their prognostic relevance using Cox proportional hazards analysis (Figure 5B) in the TCGA-BRCA cohort.

Among the biosynthetic enzymes, *ODC1* mRNA expression showed modest but significant positive correlations with both *MKI67* (rho = 0.183, *p* = 0.017) and *MCM2* (rho = 0.193, *p* = 0.012) within the basal subtype (Figure 5A). *ATP13A3* mRNA levels demonstrated strong subtype-specific associations, showing a robust positive correlation with *MKI67* (rho = 0.51, *p* < 0.0001) and a weaker positive correlation with *MCM2* (rho = 0.186, *p* = 0.015) in basal tumors. In contrast, *SMOX* (*MKI67*: rho = −0.345, *p* = 0.002; *MCM2*: rho = −0.347, *p* = 0.002) and *ATP13A4* (*MKI67*: rho = −0.235, *p* = 0.039; *MCM2*: rho = −0.285, *p* = 0.012) mRNA expression exhibited the strongest negative correlations, specifically within the HER2-enriched subtype. *SAT1* mRNA also showed a modest negative correlation with proliferation in luminal A tumors. Other genes showed weaker or inconsistent associations across subtypes (Figure 5A).

To evaluate the prognostic relevance of polyamine-related genes, we performed univariable Cox proportional hazards analyses for overall survival. In TCGA-BRCA, *ATP13A3* mRNA expression was associated with a significantly increased risk of death (HR = 1.32, *p* = 3.19 × 10^−2^), indicating an adverse prognostic effect. In contrast, *ATP13A2* (HR = 0.73, *p* = 1.52 × 10^−2^), *SMOX* (HR = 0.84, *p* = 3.42 × 10^−2^), and *PAOX* (HR = 0.69, *p* = 1.52 × 10^−3^) mRNA levels were associated with improved survival. These associations were largely retained in multivariable models adjusting for age, stage, and PAM50 subtype: *ATP13A3* remained an adverse prognostic marker (HR = 1.41, *p* = 1.25 × 10^−2^), while *ATP13A2* (HR = 0.76, *p* = 4.82 × 10^−2^), and *PAOX* (HR = 0.62, *p* = 3.90 × 10^−5^) continued to show significant protective effects (Figure 5B).

Overall, polyamine-related genes show distinct relationships with proliferation and clinical outcome. Notably, *ATP13A3* mRNA expression correlates positively with the proliferation markers, *MKI67* and *MCM2*, and exerts an adverse prognostic effect on overall survival that is independent of age, subtype and stage, consistent with a more proliferative and clinically unfavorable tumor phenotype. Conversely, *ATP13A2* exerts a protective prognostic effect on overall survival, highlighting an opposite role of *ATP13A2* and *ATP13A3* on breast cancer clinical outcomes.

### 3.4. Polyamine-Related Genes Are Enriched in Key Oncogenic Pathways

**Figure 6:**
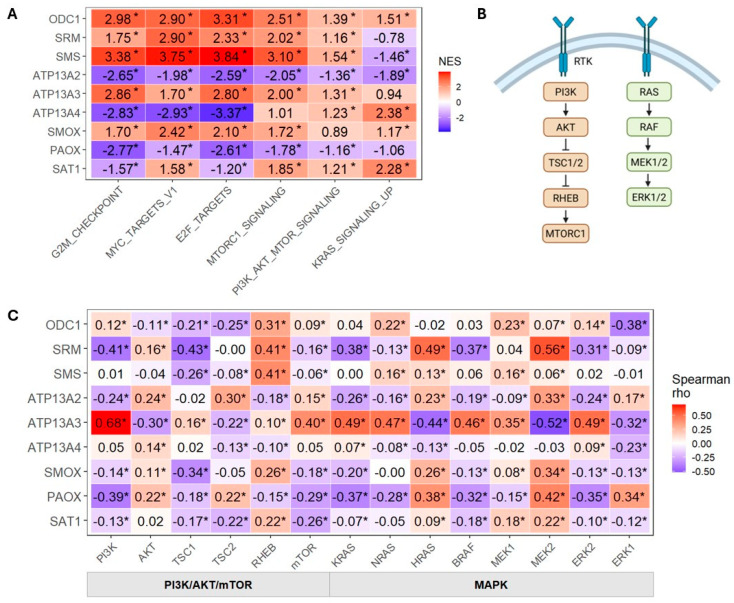
Polyamine-related genes are associated with key oncogenic pathways. (**A**) Normalized enrichment scores (NES) from gene-set enrichment analysis (GSEA) for selected Hallmark pathways comparing high versus low expression groups for each polyamine-related gene (median cutoff) in TCGA-BRCA primary tumors. Asterisk (*) indicate statistical significance (FDR < 0.25, *p* < 0.05). (**B**) Simplified schematic of the PI3K/AKT/mTOR and MAPK signaling pathways, indicating key nodes included in the correlation analyses shown in (**C**). Created in BioRender. van Veen, S. (2026) https://BioRender.com/vicf1jq. (**C**) Spearman correlation matrix showing associations between polyamine-related genes and key components of the PI3K/AKT/mTOR (left) and MAPK (right) signaling pathway across all TCGA-BRCA tumors. Colors represent the correlation coefficient (rho), and asterisks (*) indicate statistically significant correlations (*p* < 0.05).

To gain insight into the biological processes associated with polyamine-related genes in breast cancer, we performed gene set enrichment analysis (GSEA) using TCGA-BRCA transcriptomic data (Figure 6).

The biosynthetic enzymes *ODC1*, *SRM* and *SMS* were consistently enriched in proliferative transcriptional programs, including E2F targets, MYC targets, and G2M checkpoint, as well as in PI3K/AKT/MTOR and mTORC1 signaling. Among the polyamine transporters, *ATP13A3* showed a similar enrichment profile, whereas *ATP13A2* displayed inverse associations with these pathways. *ATP13A4* showed negative enrichment in G2M checkpoints, MYC targets, and E2F targets, and positive enrichment in PI3K/AKT/MTOR, and MAPK signaling. For the catabolic enzymes, *SMOX* demonstrated positive enrichment across these oncogenic programs, while *PAOX* exhibited negative enrichment (Figure 6A).

Given the enrichment of polyamine-related genes in PI3K/AKT/mTOR and MAPK signaling pathways, we next examined correlations between polyamine-related genes and key components of these pathways using TCGA-BRCA transcriptomic data (Figure 6B,C). *ATP13A3* showed consistent positive correlations with *PIK3CA*, *RHEB* and *MTOR,* alongside modest but statistically significant negative correlations with *AKT1* and *TSC2*. In contrast, *ATP13A2* exhibited negative correlations with *PIK3CA* and *RHEB*, and positive correlations with *AKT1* and *TSC2*. In addition, *ATP13A3* displayed consistent positive correlations with multiple MAPK pathway components, including *KRAS*, *NRAS*, *BRAF*, *MEK1*, and *ERK2,* and negative correlations with *HRAS*, *MEK2*, and *ERK1.* For these MAPK-associated genes, *ATP13A2* again showed correlations of opposite direction (Figure 6C). Together, these data suggest that *ATP13A2* and *ATP13A3* associate with distinct transcriptional states linked to PI3K/AKT/mTOR and MAPK signaling.

## 4. Discussion

The present study provides a systematic characterization of polyamine homeostasis across major breast cancer subtypes by integrating cell line profiling with transcriptomic analyses of large patient cohorts. Our findings reveal pronounced subtype-dependent differences across polyamine biosynthesis, catabolism, and transport, and identify ATP13A3 as a clinically relevant polyamine transporter associated with proliferative signaling and adverse outcome, particularly in basal-like breast cancer.

### 4.1. Subtype-Dependent Opposing Expression Patterns and Prognostic Significance of ATP13A2 and ATP13A3 in Breast Cancer

Polyamine transport is emerging as a critical but understudied determinant of breast cancer biology. Prior work established ATP13A4 as a putative driver of elevated PTS activity in luminal A MCF7 cells [13], yet its broader relevance across breast cancer subtypes remained largely unexplored. Our current analysis demonstrates marked heterogeneity among P5B-ATPases across breast cancer models. At the cell line level, *ATP13A4* mRNA expression was strongly upregulated in luminal B BT474 cells, consistent with enhanced PTS activity, yet was minimally detectable in HER2-enriched and basal-like cell lines. In contrast, patient-derived tumors showed significant downregulation of *ATP13A4* mRNA in both luminal and basal-like subtypes, highlighting important limitations of in vitro models in capturing subtype-specific regulation of polyamine transport. These discrepancies likely reflect regulatory influences present in the tumor microenvironment, including hormonal signaling, stromal interactions, differentiation state, and proliferative pressure, that are absent in long-term cell culture. Thus, while cell lines provide a reductionist framework to interrogate individual transport mechanisms, patient tumors reveal subtype-aligned transporter regulation that is more directly relevant to clinical behavior, providing a more informative framework for understanding subtype-specific polyamine homeostasis in breast cancer. This observation is consistent with prior reports demonstrating profound molecular differences between commonly used ovarian cancer cell lines and high-grade serous ovarian cancer tumor samples [33].

Among the P5B-ATPases, ATP13A3 displayed the most consistent and clinically meaningful pattern across datasets. *ATP13A3* transcript levels were elevated in multiple breast cancer cell lines relative to the non-tumorigenic epithelial cell line MCF10A, and were significantly increased in luminal B, HER2-enriched, and basal-like patient-derived tumors. Notably, *ATP13A3* mRNA expression strongly correlated with proliferation markers specifically in basal-like breast cancer and emerged as an independent adverse prognostic marker. While these subtype-associated patterns were robust at the transcript level, analysis of mass spectrometry-based proteomic data from the CPTAC breast cancer cohort did not reveal corresponding subtype-specific differences in ATP13A3 protein abundance, indicating that transcriptional stratification is not necessarily mirrored at the level of steady-state bulk tumor proteomics. Together, these findings suggest that ATP13A3-mediated polyamine import may amplify proliferative or metabolic programs that are particularly prominent in aggressive tumor states, potentially through regulatory mechanisms not captured by total protein abundance alone. This interpretation aligns with findings in other cancers, where ATP13A3 is enriched in highly metastatic pancreatic cancer cells [4], and associates with poor outcome in neuroblastoma cohorts [7], supporting its role as a context-general oncogenic transporter.

Conversely, ATP13A2 exhibited a more favorable clinical profile. Although *ATP13A2* mRNA expression varied across breast cancer cell lines without clear subtype specificity, patient-derived tumors showed a significant enrichment of *ATP13A2* in luminal A breast cancer and a protective association with overall survival, consistent with observations in the METABRIC breast cancer cohort [34], and in neuroblastoma cohorts [7]. Notably, a study aimed at identifying subtype-specific transcription factors regulating polyamine metabolism genes reported increased ATP13A2 expression in basal-like breast cancer subtypes [35]. This apparent discrepancy may reflect differences in subtype stratification strategies: that study assigned molecular subtypes using Genefu, a bioinformatics framework based on published gene-expression signatures [36], whereas the present analysis relied on PAM50 subtype annotations available in the TCGA-BRCA metadata. High ATP13A2 expression has also been shown to be associated with poor overall survival in cervical cancer [37], underscoring cancer-type dependent implications of polyamine transporters in tumor biology. Mechanistically, ATP13A2 functions as a lysosomal polyamine exporter that prevents lysosomal polyamine accumulation and contributes to organelle integrity and stress resilience [2]. Although the determinants of ATP13A2 expression in breast cancer remain unclear, its enrichment in luminal A tumors and association with improved survival suggest that ATP13A2 marks a less aggressive tumor phenotype.

Beyond transcriptional regulation of polyamine transporters, transport activity may also be shaped by membrane organization and intracellular trafficking. Consistent with tumor-associated membrane remodeling, CAV1 expression was broadly reduced across breast cancer subtypes relative to normal breast tissue at both the transcript and protein level. However, we did not observe a consistent association between CAV1 and ATP13A3 in bulk tumor datasets, suggesting that any functional interplay between membrane organization and polyamine transport is likely context-dependent and not readily captured by steady-state expression analyses.

### 4.2. Subtype-Dependent Metabolic Rewiring of the Polyamine Pathway

Our analyses further demonstrate that polyamine biosynthesis and catabolism are reconfigured in a subtype-dependent manner in both breast cancer cell lines and patient tumors. At the cell line level, we observed significant variation in SRM, SMS, and SMOX levels, whereas ODC1 remained relatively stable across models. In contrast, patient-derived tumors exhibited clear subtype-specific differences in *ODC1* mRNA expression, with significantly elevated levels in basal-like tumors and reduced expression in luminal subtypes, in line with previous findings [35].

Beyond *ODC1*, basal-like breast cancers showed significantly higher expression of *SMS* and *SMOX*, in line with previous findings [35], indicative of a more dynamic polyamine metabolic state. Conversely, luminal tumors displayed broad reductions in *SRM*, *SAT1*, and *SMOX*, reflecting more restrained polyamine degradation. These subtype-dependent patterns align with earlier reports showing upregulation of ODC1, and downregulation of SMOX and PAOX in breast cancer [15,16] but extend these observations by demonstrating that such changes are not uniform across all subtypes.

Importantly, polyamine pathway-level analysis revealed that subtype-specific differences in polyamine metabolism are coordinated at the level of biosynthesis and catabolism, but not transport. Whereas luminal tumors showed concerted suppression of both synthetic and catabolic programs and basal-like tumors exhibited elevated biosynthetic activity, polyamine transport did not display coordinated transcriptional regulation at the pathway level. This dissociation suggests that, unlike metabolic enzymes, polyamine transport is not governed by a unified transcriptional program but instead reflects selective regulation of individual transporters, possibly as a function of their distinct biological roles. In this context, the strong subtype specificity and clinical relevance of ATP13A3 point to a gene-specific role for polyamine uptake in aggressive breast cancer, which has been shown to have significantly higher levels of polyamines, including putrescine and spermidine, using metabolomic data of patient-derived breast cancer samples [35].

### 4.3. ATP13A3, in Contrast to ATP13A2, Associates with PI3K/AKT/mTOR and MAPK Signaling in Breast Cancer

Although multiple polyamine biosynthetic and catabolic enzymes were associated with proliferative transcriptional programs, ATP13A3 emerged as the only polyamine transporter displaying a concordant association with growth-related signaling pathways, strong subtype specificity, and an adverse clinical outcome. Gene set enrichment analysis revealed that high *ATP13A3* mRNA expression was consistently enriched in proliferative transcriptional programs, including E2F targets, MYC targets, and G2M checkpoint, as well as in PI3K/AKT/mTOR signaling. These findings are consistent with prior studies showing that polyamines can potentiate mTORC1 activity and that combined inhibition of polyamine biosynthesis and mTOR signaling results in enhanced cytotoxicity and translational suppression in breast cancer cells [17]. Moreover, spermidine has been shown to act downstream of PI3K/AKT in colorectal cancer cells, reinforcing this pathway to sustain proliferation [38].

In addition to PI3K/AKT/mTOR signaling, our analyses revealed that polyamine-related genes are also transcriptionally linked to MAPK pathway signaling. Correlation analyses demonstrated moderate and statistically significant associations between multiple polyamine genes and key MAPK components, including *KRAS*, *NRAS*, *BRAF*, *MEK1*, and *ERK2*. Notably, *ATP13A3* showed positive correlations with several MAPK pathway nodes, whereas *ATP13A2* and a subset of polyamine metabolic enzymes exhibited correlations of opposite direction. These findings indicate that polyamine homeostasis interfaces with MAPK signaling in a gene-specific and directionally divergent manner. In line with this observation, the anti-invasive effects of the ODC1 inhibitor difluoromethylornithine (DFMO) have been causally linked to MAPK pathway activation through ERK phosphorylation [21], supporting functional crosstalk between polyamine metabolism and MAPK signaling.

Notably, in head and neck squamous cell carcinoma, high ATP13A3 expression has been reported to associate with enrichment of Aurora kinase–related gene expression signatures, which are themselves characteristic of highly mitotic states and increased genomic instability [39], underscoring the cancer-type-specific consequences of altered polyamine transport. Together, these findings indicate that ATP13A3 upregulation occurs within transcriptional contexts marked by elevated growth signaling and cell-cycle progression.

Furthermore, our data reveal a strong positive correlation between *ATP13A3* and *PIK3CA*, one of the most frequently mutated genes in breast cancer [40]. Although the mechanistic basis of this association remains to be determined, it suggests that tumors with high *ATP13A3* expression may engage growth-factor–driven signaling networks, including PI3K/AKT/mTOR and MAPK pathways, more robustly, either through metabolic coupling or through broader transcriptional programs that co-regulate transport and proliferative pathways. In line with this interpretation, *ATP13A3* also correlated positively with *MKI67* and *MCM2* specifically in basal-like tumors, further supporting its association with aggressive proliferative states. Conversely, gene set enrichment analysis revealed a striking opposite pattern for *ATP13A2*, which was negatively enriched in proliferative transcriptional programs and in upstream PI3K/AKT/mTOR- and MAPK-associated signaling signatures, and negatively correlated with *PIK3CA*. These data further highlight opposing roles of ATP13A2 and ATP13A3 in breast cancer biology. *ATP13A4* displayed a distinct profile, aligning with *ATP13A2* in its negative association with proliferative transcriptional programs while exhibiting a divergent pattern across PI3K/AKT/mTOR- and MAPK-linked signaling pathways.

Together, these findings position ATP13A3 at the intersection of polyamine transport and major oncogenic signaling axes. While additional mechanistic studies are needed to define the directionality and causal relationships, the consistent enrichment of *ATP13A3* in PI3K/AKT/mTOR- and MAPK-linked transcriptional programs suggests that ATP13A3 may contribute to the metabolic and proliferative wiring that characterizes aggressive breast cancer subtypes.

### 4.4. Therapeutic Implications of Divergent Polyamine Programs Across Breast Cancer Subtypes

Our findings argue against a “one-size-fits-all” approach to polyamine-directed therapy in breast cancer. A central strategy under investigation is inhibition of polyamine biosynthesis using the ODC1 inhibitor difluoromethylornithine (DFMO). Basal-like tumors, characterized by elevated polyamine biosynthesis, may represent a more polyamine-dependent state and therefore may be more susceptible to ODC1 inhibition. By contrast, luminal tumors exhibit reduced biosynthetic activity and a globally restrained polyamine metabolic program, suggesting DFMO monotherapy may be less consistently effective in this context.

Existing data from cell models support subtype-associated differences but also reveal complexity. DFMO induced greater cell death in multiple triple-negative breast cancer cell lines compared to non-triple negative cell models [41], yet other studies reported higher DFMO sensitivity in luminal A MCF7 cells than in basal-like MDAMB231 cells [42]. Together, these findings suggest that DFMO response is influenced not only by subtype assignment but also by additional determinants (e.g., baseline polyamine pools, compensatory uptake capacity, growth-factor wiring, and culture conditions). They also reinforce the broader point that cell line behavior does not always recapitulate the regulation observed in tumors.

A clinically relevant consideration is that cancer cells can compensate for polyamine biosynthesis inhibition by upregulating polyamine uptake. In neuroblastoma, ATP13A3 mediates compensatory uptake following DFMO treatment [7]. In this framework, the opposing expression patterns, prognostic associations, and pathway correlations of ATP13A2 and ATP13A3 observed here suggest fundamentally different roles in breast cancer biology and raise the possibility that ATP13A3-high tumors may be particularly capable of maintaining polyamine supply under biosynthetic stress. Therefore, selective targeting of ATP13A3, especially in basal-like contexts where ATP13A3 is highest and prognostically adverse, could represent a more subtype-aligned strategy than broad inhibition of multiple transport isoforms using nonselective transport inhibitors. Determining whether ATP13A3 is required for compensatory uptake in breast cancer, and whether its inhibition potentiates DFMO efficacy, will be important priorities for future mechanistic work.

Together, these observations highlight the importance of molecular subtype stratification when considering polyamine-directed therapeutic strategies in breast cancer and underscore the need for mechanistic studies to define how individual components of the polyamine network contribute to tumor behavior in distinct biological contexts.

## 5. Conclusions

This study reveals that polyamine homeostasis varies substantially across breast cancer subtypes. By integrating cell-line profiling with patient tumor transcriptomics, we show that both polyamine transport and metabolism follow subtype-specific patterns with clinical relevance. ATP13A3 consistently associates with proliferative signaling and poor survival, whereas ATP13A2 aligns with more favorable outcomes, highlighting opposing roles for these transporters in breast cancer. Polyamine metabolic enzymes likewise exhibit distinct patterns in basal-like versus luminal tumors.

These findings demonstrate that polyamine regulation is an important contributor to breast cancer heterogeneity and suggest that therapeutic strategies targeting this pathway will require subtype-specific consideration. Further mechanistic studies will be necessary to define how individual polyamine regulators interface with oncogenic programs and whether modulating polyamine homeostasis can be leveraged therapeutically.

## Figures and Tables

**Table 1:** Gene accession numbers and primer sequences.

Gene	NCBI Accession Number	Primer Sequences
*GAPDH*	NM_002046.7	F: GTCTCCTCTGACTTCAACAGCG R: ACCACCCTGTTGCTGTAGCCAA
*ATP13A2*	XM_005245810	F: CATGGCTCTGTACAGCCTGA R: CTCATGAGCACTGCCACTGT
*ATP13A3*	XM_047448904	F: TACTGTGGAGCACTGATG R: GAGTTGCCACCATGTCATGC
*ATP13A4*	XM_047449063	F: CCAGCACGCTCTGCTCAATG R: GAAGATGGATCCGGCAAGGC

**Table 2:** Breast cancer cell lines grouped by breast cancer subtype.

Luminal A	BT483, CAMA1, EFM19, HCC1428, HCC1500, KPL1, MCF7, MDAMB134VI, MDAMB175VII, MDAMB415, T47D, ZR751
Luminal B	BT474, EFM192A, HCC1419, MDAMB361, SUM44PE, SUM52PE, SUM52, UACC812, ZR7530
HER2-enriched	AU565, HCC1569, HCC1954, HCC202, HCC2218, JIMT1, MDAMB453, SKBR3, SUM190PT, UACC893
Basal-like	BT20, BT549, CAL120, CAL148, CAL51, CAL851, Du4475, HCC1143, HCC1187, HCC1395, HCC1599, HCC1806, HCC1937, HCC2157, HCC38, HCC70, HDQP1, HMC18, Hs 578T, MDAMB157, MDAMB231, MDAMB436, MDAMB468, MFM223, SUM102PT, SUM1315MO2, SUM149PT, SUM159PT, SUM185PE, SUM229PE

## Data Availability

All analysis scripts and raw data supporting the findings of this study will be made publicly available upon publication through publicly accessible repositories (Zenodo (version 0.1, https://doi.org/10.5281/zenodo.18442149).

## Supplementary Materials

The following supporting information can be downloaded at https://www.mdpi.com/article/10.3390/biom16020255/s1: Figure S1: *CAV1* (caveolin-1) expression across CCLE, TCGA and CPTAC breast cancer datasets. Figure S2: CPTAC mass spectrometry-based protein expression of ATP13A2, ATP13A3, and ATP13A4 across breast cancer subtypes. Figure S3: Uncropped Western blots used in Figure 3B.

## Abbreviations

The following abbreviations are used in this manuscript:

PUT	Putrescine
SPD	Spermidine
SPM	Spermine
DFMO	Difluoromethylornithine
CCLE	Cancer Cell Line Encyclopedia
