# Supplementary material for: Opposing Roles for ATP13A2 and ATP13A3 in Breast Cancer Subtype-Specific Polyamine Homeostasis
Source: Biomolecules. 2026 Feb 5;16(2):255. doi: 10.3390/biom16020255 (PMC12938054; doi:10.3390/biom16020255)
Supplement: Supplementary file 1 [file biomolecules-16-00255-s001.zip › biomolecules-4102993-supplementary.pdf]

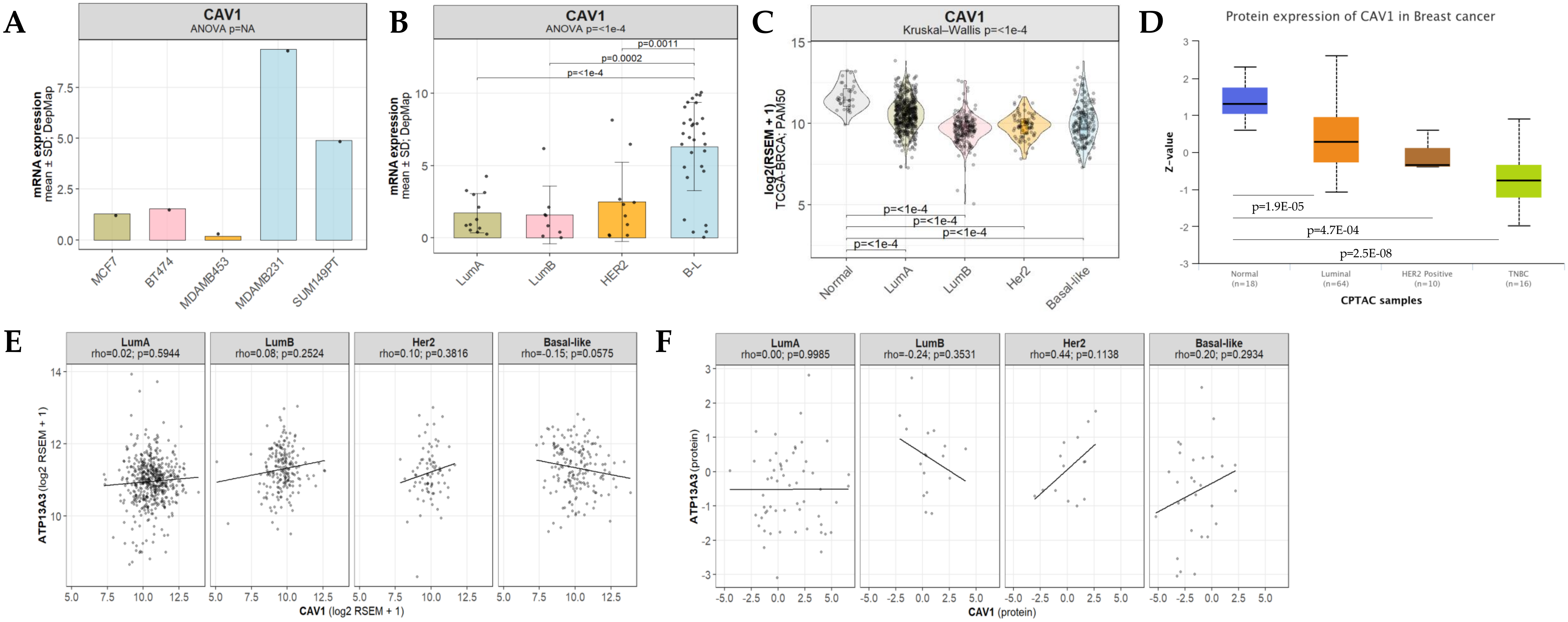

Figure S1: *CAV1* (caveolin-1) expression across CCLE, TCGA and CPTAC breast cancer datasets. (A) *CAV1* mRNA expression in selected breast cancer cell lines extracted from the Cancer Cell Line Encyclopedia (CCLE) dataset. **(B)** CCLE transcriptomic expression of *CAV1* stratified by breast cancer molecular subtype [Luminal A (LumA) n = 12, Luminal B (LumB) n = 9, HER2-enriched (HER2) n = 10, Basal-Like (B-L) n = 30 (Table 2)]. Statistical significance was determined by one-way ANOVA with Tukey HSD post-hoc test. Significant p-values ($p < 0.05$) are indicated. Data are presented as mean ± SD. **(C)** *CAV1* mRNA expression across PAM50 breast cancer subtypes [Normal-like n = 36, LumA n = 499, LumB n =197, HER2 n = 78, B-L n = 171] extracted from the Breast Invasive Carcinoma (TCGA, PanCancer Atlas) dataset accessed via cBioPortal. **(D)** CAV1 protein abundance across breast cancer subtypes in the CPTAC cohort. Z-score–normalized mass spectrometry–based protein expression values were retrieved from the UALCAN portal, with sample sizes indicated. Statistical significance was evaluated using Welch's t-test (UALCAN interface). **(E-F)** Spearman correlation analysis between *CAV1* and *ATP13A3* expression across PAM50 breast cancer subtypes using TCGA transcriptomic data (**E**) and CPTAC proteomic data (**F**), obtained via cBioPortal. Spearman's rho and corresponding p-values are indicated in each panel.

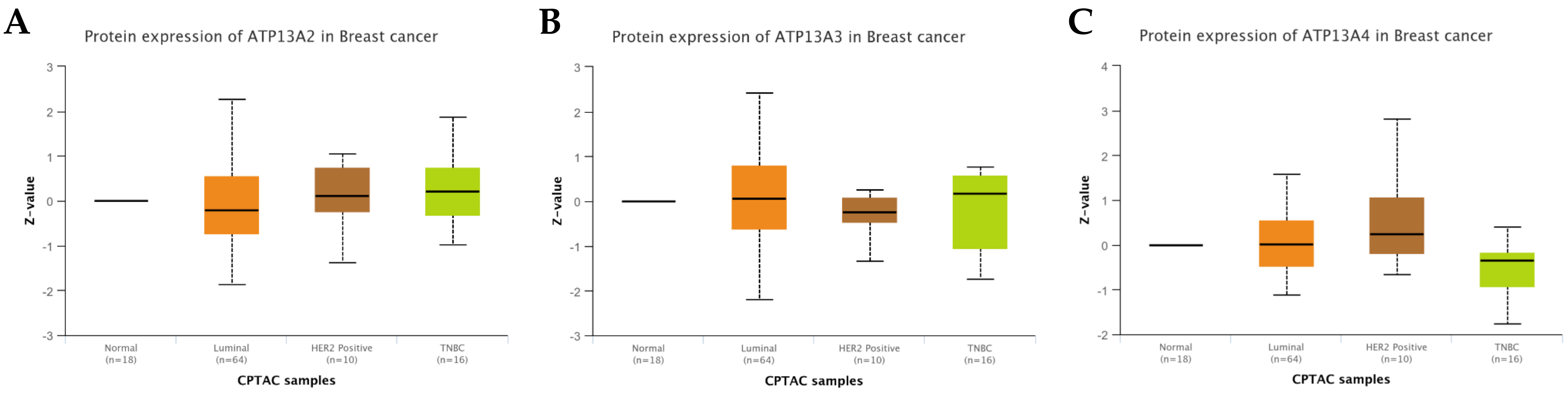

Figure S2: CPTAC mass spectrometry-based protein expression of ATP13A2, ATP13A3, and ATP13A4 across breast cancer subtypes. Protein abundance of ATP13A2 **(A)**, ATP13A3 **(B)**, and ATP13A4 **(C)** in primary breast cancer tumors from the CPTAC cohort, quantified by antibody-independent mass spectrometry. Protein levels are shown as z-score normalized values across samples and stratified by molecular subtype. No statistically significant p-values were obtained. Data were obtained via the UALCAN portal.

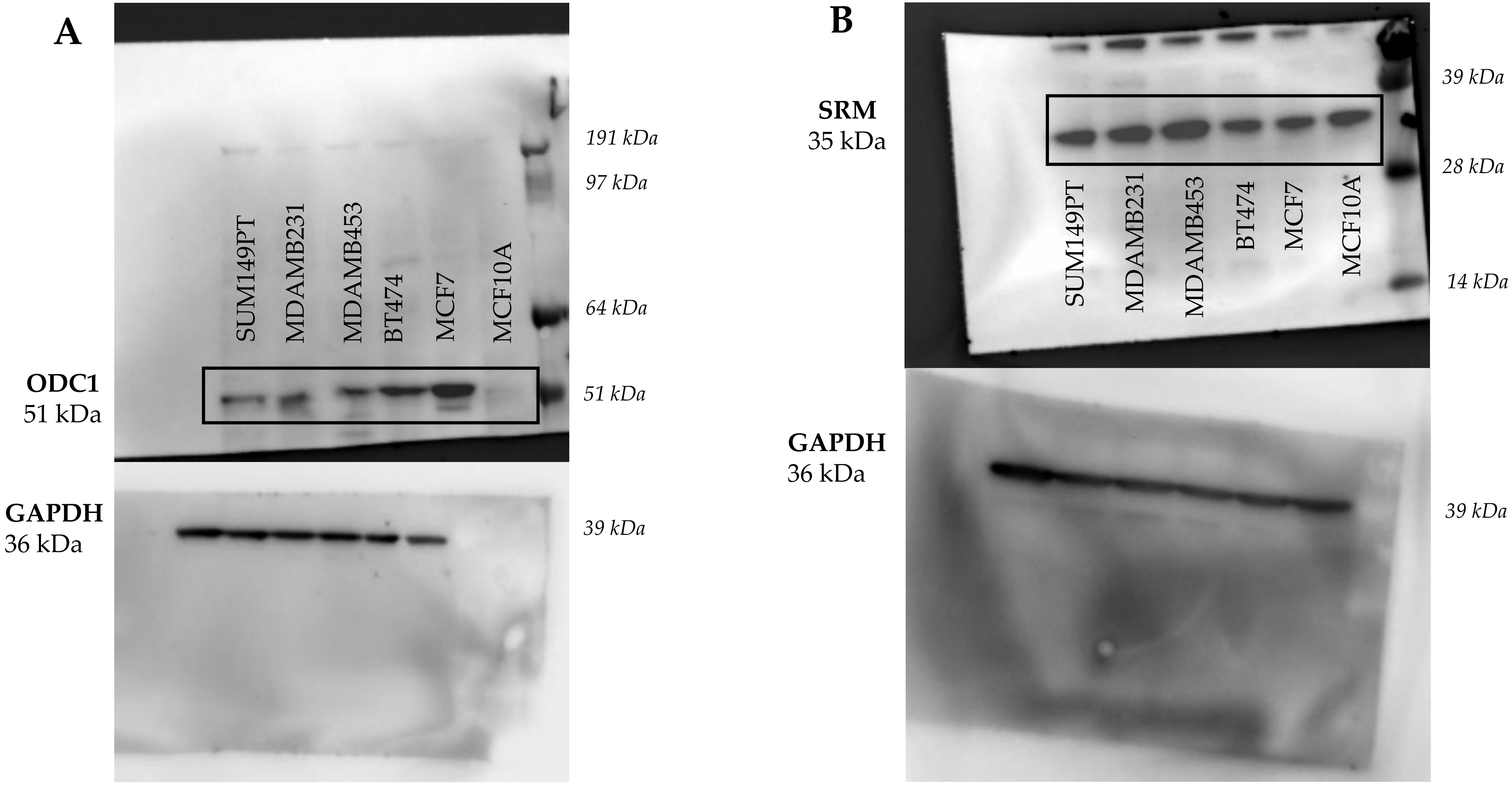

Figure S3: Uncropped Western Blots used in Fig. 3B. (A) Anti-ODC1 with GAPDH. **(B)** Anti-SRM with GAPDH.

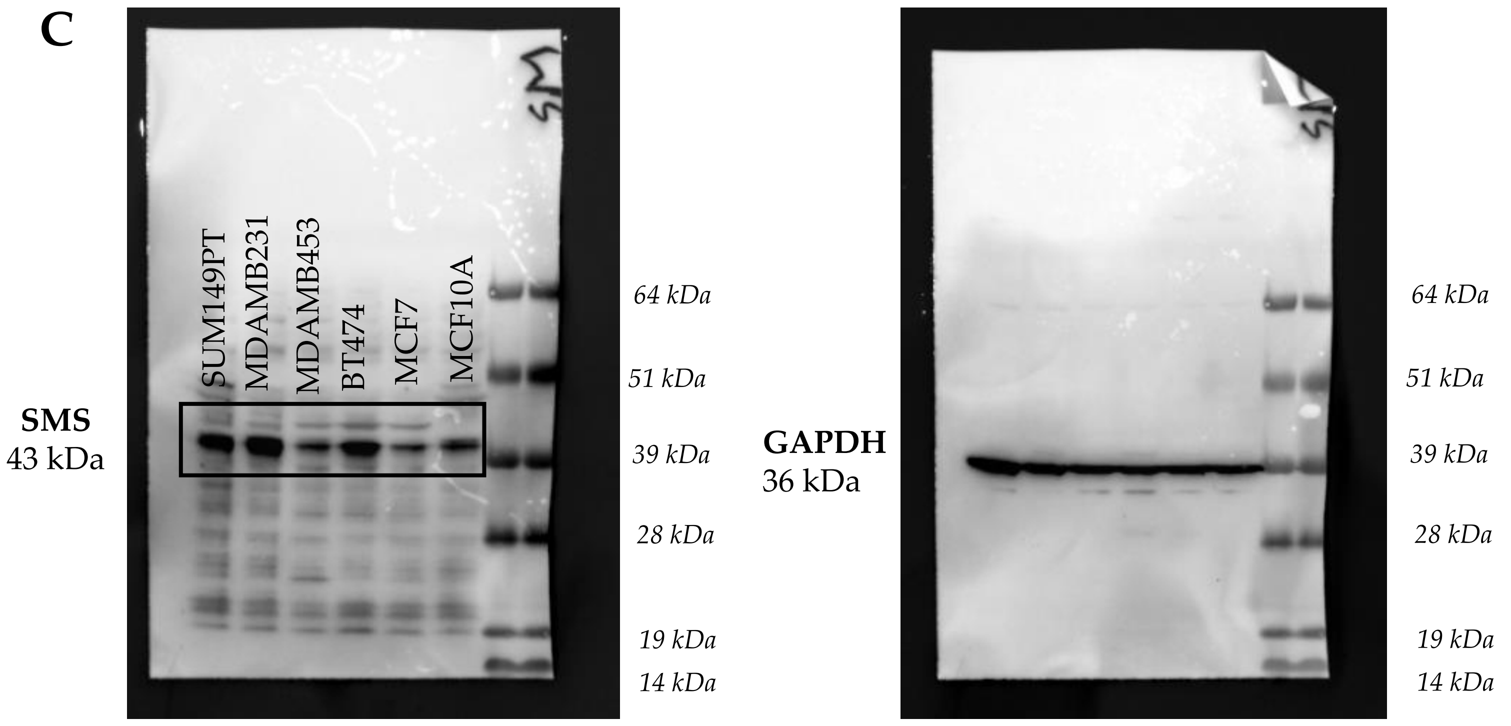

Figure S3: Uncropped Western Blots used in Fig. 3B. (C) Anti-SMS with GAPDH.

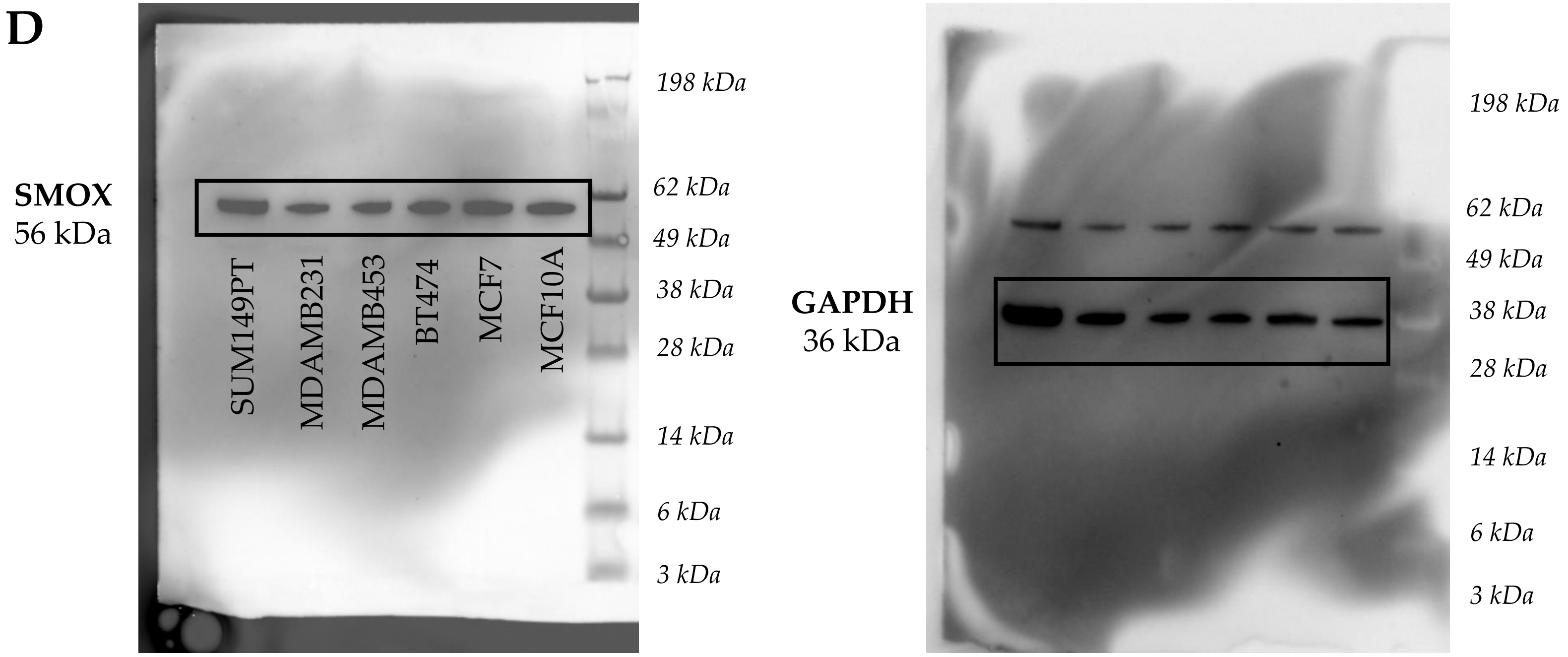

Figure S3: Uncropped Western Blots used in Fig. 3B. (D) Anti-SMOX with GAPDH.
